# Beef supply chains and the impact of the COVID-19 pandemic in the United States

**DOI:** 10.1093/af/vfaa054

**Published:** 2021-02-05

**Authors:** Derrell Peel

**Affiliations:** Department of Agricultural Economics, Oklahoma State University, Stillwater, OK, USA

**Keywords:** beef supply chains, COVID-19, food service, retail grocery

**Implications**

- This article describes the initial impacts of COVID-19 on beef supply chains and beef product markets. An overview of major beef market segments of retail grocery, food service, and exports is provided, including a focus on ground beef markets.
- COVID-19 caused massive and unprecedented impacts on beef supply chains for food service and retail grocery.
- The shutdown of food service and the corresponding surge in retail grocery demand provoked a first wave of product shortages due to bottlenecks and rigidities in food service and retail grocery supply chains.
- A second wave of impacts resulted from COVID-19-induced reductions in cattle slaughter and beef production. This resulted in additional product shortages in retail grocery.
- COVID-19 revealed both the efficiencies and rigidities of specialized food service and retail grocery supply.
- Dramatic and varied market price reactions across beef product markets helped rebalance markets and restore equilibrium.

## Introduction

One of the many factors that make the U.S. cattle and beef industry an extremely complex set of markets is the disassembly of the beef animal into thousands of different products. These products are marketed in a vast array of final markets including retail grocery, food service and exports (Clark, 2019). In the United States, food expenditures prior to COVID-19 consisted of two, roughly equal market channels: food service, representing 54% of expenditures, and retail grocery, representing 46% of expenditures (USDA-ERS, 2020). The unprecedented impacts of COVID-19 revealed to producers, processors and consumers, efficiencies as well as vulnerabilities of beef industry supply chains. The COVID-19 impacts occurred in two different but overlapping waves beginning in mid-March 2020 with the majority of impacts over by late-June. However, economic ripples continued for many weeks thereafter.

Beef packers provide the animal harvest and the primary fabrication of beef carcasses into wholesale products. Typically, packers fabricate several hundred basic wholesale products, which are marketed as several thousand products representing unique customer specifications. Subsequently, the majority of wholesale beef products move through a diverse and specialized set of further processing activities that further expand the set of products by several thousand additional products into largely separate supply chains. Following sections provide a brief overview of beef market sectors to better understand the impacts of COVID-19 on beef markets.

## Retail Grocery Sector

Retail grocery is responsible for a large amount of beef sales and grocery sales are generally recognized as the main driver of total beef sales. Retail grocery typically sells a core set of products that is rather broad but also has considerable flexibility to adjust and feature different products when market conditions are favorable. Many supermarkets no longer have butchers or do any meat cutting in the store. Some independent stores and at least one regional grocery chain are exceptions to this. The majority of supermarkets receive case-ready product from further processors, many of which are owned by major packing companies. Further processing for retail grocery involves cutting, packaging, and labeling for retail, including ground beef retail packaging. Ground beef is discussed separately in more detail below. Retail grocery increasingly may include premarinated, ready to cook “meal kits” or similar value-added products. Retail grocery uses almost entirely fresh beef products with beef features following a standard calendar in which advertising and purchasing are planned several weeks to several months in advance.

## Food Service Sector

Food service includes facilities sometimes referred to as HRI, meaning hotels, restaurants, and institutions (hospitals, schools, etc.). Restaurants represent a wide range of establishments including quick service restaurants (QSR or fast food), fast casual, cafeterias/buffets, casual dining, midlevel, and fine dining. Individual restaurants or chains typically have limited and fixed beef product needs that are very specific and quite rigid. Collectively, restaurants utilize a wide range of beef products from ground beef to Prime steaks. The majority of food service beef products originate from further processing facilities in which products are trimmed, sized for portions, tenderized, marinated or otherwise processed according to specifications. Food service further processing often produces additional beef products as primals and subprimals are further fabricated into multiple products including bench trim that is used for cooked ingredients in other processed products. Although most food service facilities use fresh beef products, some restaurants may utilize frozen portion-control steaks or other products that can be thawed on demand.

## Ground Beef

Ground beef represents an estimated 45% of total U.S. beef consumption (Ishmael, 2020) and plays a singular and uniquely important role in the U.S. beef industry in both retail grocery and food service sectors. Retail grocery establishments market large quantities of ground beef in a variety of forms and packaging. Ground beef for retail grocery is commonly part of supply chains that specialize in case ready products and processing specifically for grocery. For retail grocery, ground beef is typically made from fresh domestic meat products, frequently sourced from muscles and trimmings from specific primals as supermarkets often market ground beef with carcass references such as ground chuck, ground round, ground sirloin, etc.

Ground beef for food service is typically provided by specialized grinders that utilize a diverse set of inputs including fresh 50% (or similar) fatty trimmings,^1^ fresh lean trimmings or muscles from fed slaughter, fresh or frozen cow/bull lean trimmings, and frozen imported lean trimmings. Margins are razor thin in food service, especially in QSRs that feature dollar menus, etc. and ground beef formulation is subject to intense cost scrutiny. Though there is some potential overlap in input sources for food service and retail grocery ground beef, the resources used for each tend to be largely separate.

## Exports

Beef exports frequently originate with packers that produce export products according to unique specifications that are typically different from domestic products; or from exporting companies that may do additional fabrication/processing for export. Growing exports in recent years and expanded demand for specific products have significantly changed domestic markets. For example, various chuck products are popular in some Asian markets and have increased prices relative to other products and changed seasonal price patterns. Food service grinders, that can and have used these chuck items in ground beef now find that these products are effectively priced out of the ground beef market. Most exports are frozen and products are typically staged in cold storage prior to shipment.

## Imports

The United States has long been a major beef importing country, despite being the largest beef producing country and a major beef exporter. Beef imports are driven by the demand for lean trim to support ground beef production with over 70% of imports estimated to be processing beef. It is estimated that imported lean trimmings accounts for over 25% of the total trim used for ground beef production in the United States. Imported beef also includes some muscle cuts from Canada and Mexico. Beef trade with Canada includes some bilateral trade of similar products that are economically motivated by north–south transportation efficiencies (compared to east–west shipping in both countries). Beef imports from Mexico have grown dramatically in recent years and largely represent retail grocery and food service products targeted to Hispanic markets in the United States.

## COVID-19 Impacts

### Food service shutdown

**Figure1.**
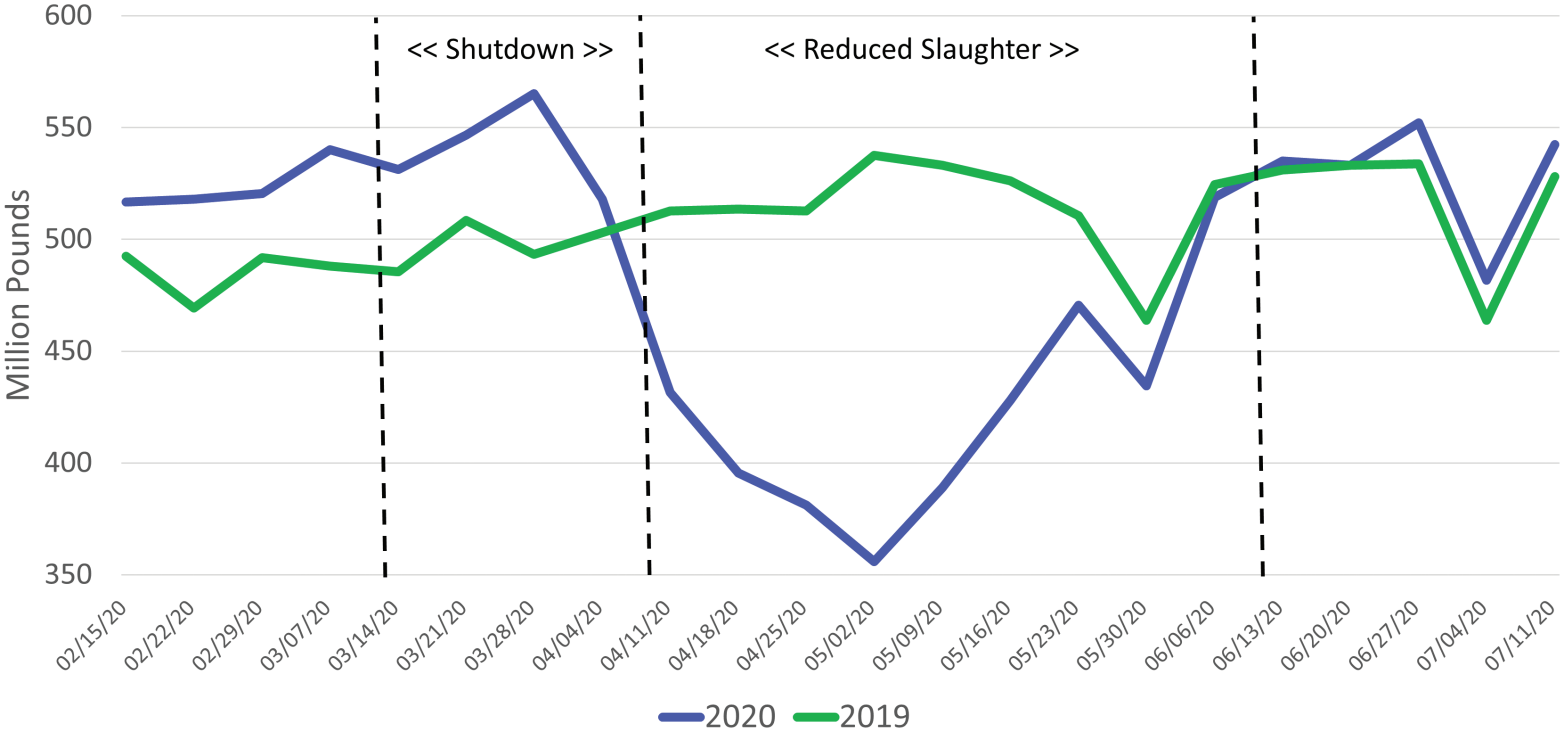
Beef production, federally inspected weekly.

The first wave of impacts, which began in mid-March, resulted from the near total shutdown of food service. Abruptly, food demand at retail grocery nearly doubled. The surge in retail grocery demand was further aggravated by panic buying as consumers attempted to stockpile food at home. Retail grocery demand quickly overwhelmed the retail grocery supply chain resulting in localized and temporary shortages in retail stores. It is important to recognize that there was no actual shortage of product during the first month of the shutdown, but rather bottlenecks in the supply chains. Figure 1 shows beef production was above year earlier levels until April. It became quickly apparent that food service and retail grocery supply chains are very specialized and as a result somewhat inflexible. Food service processors are not equipped to package and label products for case-ready retail sale; and in many cases, distribution systems are largely separate.

The shutdown affected various beef products differently according to their primary use. Table 1 shows that the initial impacts were price decreases for products heavily used in food service, for example, tenderloin (IMPS 189A),^2^ brisket (IMPS 120A) and ribeye (IMPS 112A). Simultaneously, products used at retail, especially products that support retail ground beef, that is, chuck and round products (IMPS 114A, 116A, 168, 170) saw immediate price increases (Table 1). Over a period of weeks, adjustments eventually allowed some food service processors, distributors and retailers to adapt to retail grocery demand. Creative solutions included some restaurants, experiencing no, or greatly reduced, foot traffic, selling raw product direct to consumers, either from existing inventories when the shutdown occurred, or because they still had access to food service supply chains. QSR recovered somewhat quicker than full service restaurants because of the availability of drive-thru service, which further reduced some of the retail grocery burden. Full service restaurants developed or emphasized take-out, curbside and delivery options, often with a limited menu. Adjustments to the limited food service channel continued through April and May and, to some extent, for many weeks thereafter.

**Table 1.** Wholesale beef price changes, selected products

	Product	IMPS*	Shutdown impacts	Reduced production impacts
			% Price change 3/6/20–4/10/20	% Price change 4/10/20–5/8/20
1	Ribeye	112A	–17.1	+80.9
2	Chuck Clod	114A	+27.1	+150.7
3	Chuck Roll	116A	+39.1	+67.9
4	Brisket	120A	–15.5	+105.5
5	Inside Round	168	+28.4	+106.2
6	Gooseneck Round	170	+31.1	+90.6
7	Loin Strip	180	+17.8	+50.7
8	Loin Top Butt	184	–2.1	+129.0
9	Tenderloin	189A	–42.0	+126.2
10	Fresh 50s	Trim	–38.9	+720.0

Data sources: USDA AMS LM_XB 459 and LM_XB 460, compiled by the Livestock Marketing Information Center.

*Institutional Meat Purchase Specifications.

**Figure 2.**
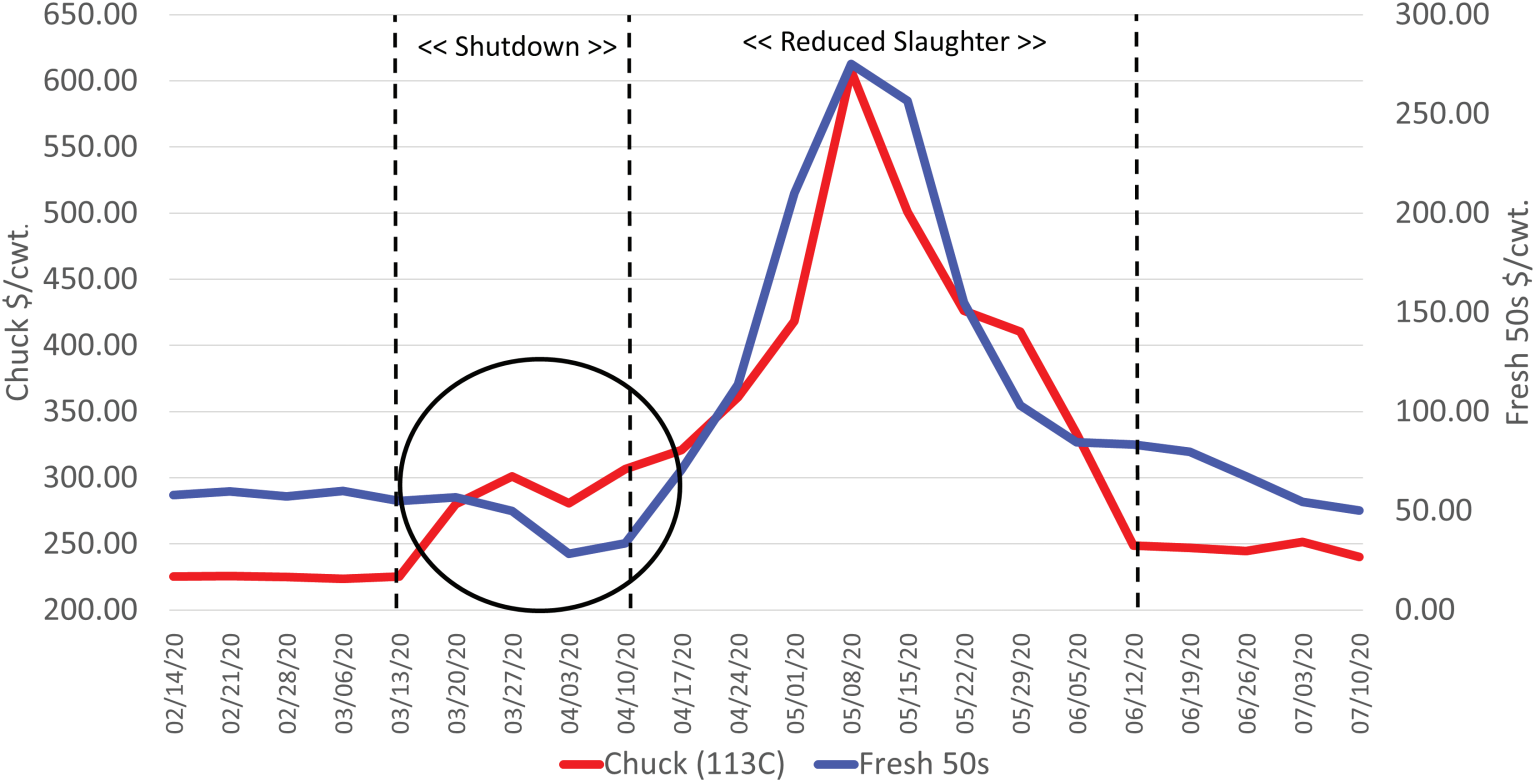
Food service vs. retail grocery ground beef demand.

The contrasting impacts in retail grocery and food service ground beef supply chains are demonstrated in Figure 2, which shows weekly prices for chucks (IMPS 113C) and fresh 50% trimmings. In the first few weeks after the shutdown of food service, markets for ground beef sources showed diverging price impacts. From early March to early April, the price of beef chuck clods (IMPS 114A) and chuck rolls (IMPS116A) increased sharply, driven by sharply higher retail grocery demand for ground beef (Table 1). Simultaneously, the price of 50% lean trimmings, used primarily for food service ground beef, decreased nearly 39%, to 18-yr lows. After another 4 wk or so, arbitrage and adjustments re-established the normal price relationships between these beef product markets. Most beef wholesale markets increased to record levels in April and May (Table 1) due to the supply disruptions in beef packing, although tenderloin (IMPS 189A) did not reach record high levels because the product is heavily dependent on food service demand (Table 2), which remained severely reduced.

**Table 2.** Wholesale beef prices (weekly), selected products ($/cwt)

Product	IMPS*	2020^†^		2019			2014–2018		
		Max	Min	Ave	Max	Min	Ave	Max	Min
Ribeye	112A	1158.75^‡c^	588.47	815.10	949.78	713.49	748.50	1031.08	550.13
Tenderloin	189A	1238.88	533.33^||^	1042.89	1428.98	920.38	1023.96	1273.21	827.36
Chuck Clod	114A	671.49^‡a^	190.75	224.18	248.34	205.58	231.14	324.12	177.13
Chuck Roll	116A	628.94^‡a^	251.00	282.93	359.03	252.05	287.82	367.37	229.52
Inside Round	168	650.35^‡b^	208.53	224.09	256.44	203.74	240.32	364.88	178.89
Fresh 50s	Trim	275.28^‡a^	28.49^$^	76.57	99.63	43.14	83.54	200.80	32.20

Data sources: USDA AMS LM_XB 459 and LM_XB 460, compiled by the Livestock Marketing Information Center.

*Institutional Meat Purchase Specifications.

^†^Year-to-date through October 2, 2020.

^‡^Record high (data series), a = week ending May 8, 2020; b = week ending May 15, 2020; c = week ending May 22, 2020.

^||^Record low (data series), week ending April 10, 2020.

^$^Low since November 2002, week ending April 3, 2020.

### Packing plant disruptions

The second wave began in April when COVID-19 affected the labor force of harvest and processing installations and severely reduced output. Never before have so many packing and processing plants been affected simultaneously by reductions in capacity. Some harvesting plants completely shut down for up to 2 wk and others curtailed output due to labor force reductions. Cattle slaughter decreased weekly through the month of April, reaching a peak reduction of 34.8% down year over year the end of April, and then slowly recovered through May. Total beef production over a 9-wk period of these effects was down 17.9% compared to the same period 1 yr earlier (Figure 1). This reduction in beef production resulted in real, though temporary, shortages of product that looked to many consumers like more of the same conditions as the initial shutdown in March and early April. The beef supply disruptions were exaggerated by the continuing limitations in the food service sector and the added demand continued to stress the retail grocery supply chain. Over several weeks, additional adjustments were made to help food service supply chains support retail grocery including more bulk packaging and, in some cases, temporary exemptions from some labeling requirements.

Retail beef prices, which reflect retail grocery prices (as opposed to food service), responded as expected but with some difference in timing. The monthly retail all-fresh beef price^3^ increased modestly in April before spiking higher in May and June. The May all-fresh price jumped to $704.50/cwt, up 18.7% over the January and February average pre-COVID level of $593.60/cwt and 19.3% higher year over year. The all-fresh retail price peaked in June at $738.20/cwt, up 26.2% compared to June 2019 levels. Retail beef prices decreased in July and August, albeit more slowly than wholesale beef prices, and remained higher year over year in August. An important market function is to use higher prices to ration demand when supplies are limited and thus avoid shortages. In this situation, the dramatic rise in retail beef prices helped to ration limited beef supplies in the March to May period but the magnitude of the shock and supply disruptions overwhelmed rising prices and led to temporary, sporadic product shortages in retail grocery.

The impact on and perception of consumers to these two waves of impacts was similar due to the lack of product in grocery stores in both cases. The reality of those impacts was very different—one due to bottlenecks and rigidities in the supply chain and the other the result of actual reductions in product availability. The combined impacts of the shutdown and packing plant disruptions were unprecedented volatility in beef product markets. Table 2 shows numerous and varied impacts on selected beef product markets. For example, the shutdown of food service caused beef tenderloin (IMPS 189A) to drop to record low levels in April, reaching a weekly low that was down 49% from the 2019 average level (Table 2). Many wholesale beef prices reached record high levels in May because of the supply shortages. Some products, such as the chuck clod (IMPS 114A) and inside round (IMPS 168) briefly reached levels nearly three times the average 2019 price for those products (Table 2). Perhaps most dramatic was the price of fresh 50s trim, which dropped to an 18-yr low in early April before jumping to a record high 5 wk later. The May price peak for fresh 50s trim was over 3.5 times higher than the average price level in 2019 (Table 2).

The focus of this article is on beef products and supply chains from packers downstream. However, upstream cattle production is broadly part of the beef supply chain and cattle markets were significantly affected by the packing plant disruptions in April and May and beyond. The reductions in packing plant operations effectively cleaved beef product markets from cattle markets for several weeks. During this period, beef product markets generally moved in opposite directions from fed cattle markets. The lack of packing capacity created beef shortages that led to immediate and dramatic price spikes for beef products while that same lack of packing capacity created an immediate excess supply of fed cattle relative to packer demand and led to lower fed cattle prices. Many concerns were voiced about the disconnect between fed cattle markets and boxed beef cutout prices when there was, in fact, an unavoidable temporary physical disconnect that caused prices to move in opposite directions above and below the packing level of the industry. Although this was an extremely unusual situation, the market reactions at all levels were exactly what is expected and helped support a remarkably rapid recovery in beef product markets.

### Continuing impacts

The initial impacts of the food service shutdown and packing plant disruptions were largely past by the end of June. Beef production returned to year earlier levels by mid-June (Figure 1). However, ripple effects, both physical and economic, continued for many weeks thereafter. By the end of June, the majority of wholesale beef product prices had returned to prior levels with typical relationships between beef product prices mostly reestablished. As noted above, retail beef prices jumped higher with some delay and reverted more slowly to lower levels as is typical of retail price adjustments.

Major impacts on fed cattle markets extended well beyond June. Reduced cattle slaughter in April and May resulted in a large backlog of fed cattle that took many weeks over the summer and fall to work through. No cattle were depopulated and delayed feedlot marketings resulted in excess supplies of fed cattle that pushed fed cattle price lower into July before recovering into the fall. Delayed fed cattle slaughter resulted in heavier carcass weights, higher quality grading percentages and other lingering impacts on beef supplies and product mixes.

Beef exports also dropped sharply in May and June before recovering in July and by August exceeded year earlier levels. Beef imports spiked higher with a delay, jumping sharply in July and remaining well above year earlier levels in August. Beef imports increased in the summer in response to strong ground beef demand from the recovery of QSR restaurants. Large supplies of fatty trimmings resulting from heavy carcass weight in domestic fed cattle and stimulated additional lean demand.

## The Role of Cold Storage

The beef production disruptions and product shortages resulting from COVID-19 provoked questions about the availability and use of beef in cold storage to supplement fresh beef supplies. Most beef marketed in food service and retail grocery is fresh and moves to final consumption in a matter of a few days to a few weeks at most. Cold storage inventories are frozen stocks of products held more than 30 d and does not typically consist of a complete set of beef products. Beef in cold storage includes products staging for export, which are mostly frozen and imported beef products (also frozen) until they are used. Cold storage beef supplies typically increase in the fall and winter and include supplies of domestic lean trimmings resulting from seasonally higher cow culling in the fall of the year. These lean trimmings are used to support ground beef production for seasonal grilling demand the following summer. Occasionally, unique market situations result in commercial firms putting other products into cold storage. This is not a regular or preferred practice as cold storage adds cost and frozen beef is often marketed at a discount to the fresh product.

In 2020, beef in cold storage increased counter-seasonally in March before declining seasonally in April and May, likely as a result of the abrupt loss of food service demand. Cold storage was not available to offset retail grocery product shortages because it consists of the wrong set of products and because the quantity of cold storage holding is small. Monthly beef cold storage inventories averaged 458 million pounds in 2019. This represents less than 1 wk of beef production, which averaged over 513 million pounds per week in 2019. This means that even if all cold storage beef was pulled out at once, it would represent less than 1 wk of beef supply and, as noted previously, would not match the mix of products needed in food service and retail grocery markets.

### Recovery and permanent impacts?

As noted previously, the majority of the initial COVID-19 shocks were resolved by the end of June. Certainly, there were continuing impacts after June and additional recovery will occur for many months. Ripple effects on beef quality, product mixes, beef exports and imports, and feedlot dynamics will extend beyond 2020. Moreover, at the time of writing in October 2020, the public health crisis and the macroeconomic impacts of COVID-19 in the United States and globally were continuing and could lead to new or renewed impacts going forward. Food service is still significantly diminished with a slow recovery that will last many more months. Clearly, recovery is a moving target as 2020 moves into the fourth quarter.

Many questions have been raised about likely permanent or very long-lasting effects of COVID-19 on food industries, beef supply chains, and the cattle industry. Given the on-going nature of the situation, it is premature to say anything definitive about permanent impacts. There are clear indications of several changes that will affect food industries; including the likely loss of food service establishments (which may rebuild or be replaced over time); changes in travel, especially business travel in a world of Zoom meetings; a larger role for take-out, drive-thru, and home delivery food service; and the extent to which home food preparation results in a long-term reduction in food service demand. Only time will provide the perspective to understand the types of changes and long-term impacts on food industries.

COVID-19 also exposed rigidities and lack of agility of beef supply chains to respond to this type of shock. Questions have been raised about the likelihood of fundamental changes to increase the resilience of beef supply chains. Certainly, it seems likely that individual firms may assess some business practices and make some changes. Firms may consider a wider range of procurement practices, such as contracts and other means to enhance both supply reliability and manage price risk. Many firms may devote additional resources to management planning with such things as supply chain mapping and other activities that will help identify supply chain redundancies and contingency plans to reduce the impact of future supply shocks. However, the current structure of food service and retail grocery supply chains evolved in response to efficiencies and economic returns to specialized facilities and have contributed to reducing the cost of food in the United States. Less specialized multifunction facilities that operate simultaneously in food service and retail grocery chains would be less efficient and more costly. Marginal changes in management and operational flexibility to increase the tactical agility of firms are more likely than massive reinvestment in new beef supply chain infrastructure.

## Summary

COVID-19 has revealed both strengths and weaknesses in beef supply chains. It has also revealed much about market economics. Under normal, stable market conditions, markets coordinate resource and product allocation with such efficiency and subtly as to be largely unrecognized. Only in the face of abrupt and unexpected shocks are the reactions of markets to rebalance and restore equilibrium revealed. Freely operating markets react with dramatic, sometimes surprising, and confusing responses to massive and unprecedented shock such as COVID-19. Consumers, producers, companies, and policymakers all learned much about how beef supply chains and the market-based economy works as a result of COVID-19.

## Footnotes

1 Fabrication of feedlot finished (fed) steer and heifer carcasses produces fatty trimmings, which are mixed with additional lean to produce ground beef. Beef packers may produce and sell a range of trimmings products with varying lean percentages but the most common are fresh 50% lean trimmings (50s).

2 Specific beef product references are identified by the corresponding Institutional Meat Purchase Specification (IMPS) product code. IMPS codes are maintained by the Agricultural Marketing Service (USDA-AMS) and are available at https://www.ams.usda.gov/sites/default/files/media/IMPS100SeriesDraft2020.pdf.

3 The all-fresh retail beef price is a composite retail grocery price series provided by the Economic Research Service (USDA-ERS) that includes a variety of Choice and other grade beef products to better reflect retail beef purchases. The data is available at https://www.ers.usda.gov/data-products/meat-price-spreads/.

## About the Author

**Derrell Peel** is the Charles Breedlove professor of agribusiness in the Department of Agricultural Economics. He has served as the extension livestock marketing specialist since he came to Oklahoma State University in 1989. He has BS and MS degrees from Montana State University and a PhD from the University of Illinois. His main program areas at Oklahoma State University include livestock market outlook and marketing/risk management education for livestock producers.

Derrell also works in the area of international livestock and meat trade with a particular focus on Mexico and Canada and the North American livestock and meat industry. He lived in Mexico on sabbatical in 2001 and has developed an extensive knowledge of the Mexican cattle and beef industry and the economics of cattle and beef trade between the United States and Mexico.
